# Comparative genomics and transcriptional profiles of *Saccharopolyspora erythraea *NRRL 2338 and a classically improved erythromycin over-producing strain

**DOI:** 10.1186/1475-2859-11-32

**Published:** 2012-03-08

**Authors:** Clelia Peano, Adelfia Talà, Giorgio Corti, Daniela Pasanisi, Miriana Durante, Giovanni Mita, Silvio Bicciato, Gianluca De Bellis, Pietro Alifano

**Affiliations:** 1 Institute of Biomedical Technologies, National Research Council, Via Fratelli Cervi 93, 20090 Segrate, Milano, Italy; 2 Dipartimento di Scienze e Tecnologie Biologiche ed Ambientali, Università del Salento, Via Monteroni, 73100 Lecce, Italy; 3 Operative Unit of Lecce, CNR - Institute of Sciences of Food Production (ISPA), via Provinciale Lecce-Monteroni, 73100 Lecce, Italy; 4 Center for Genome Research, Dept. of Biomedical Sciences, University of Modena and Reggio Emilia, Via G. Campi 287, 41100 Modena, Italy

**Keywords:** Saccharopolyspora erythraea, Secondary metabolism, Antibiotic fermentation, Strain improvement, Comparative genomics

## Abstract

**Background:**

The molecular mechanisms altered by the traditional mutation and screening approach during the improvement of antibiotic-producing microorganisms are still poorly understood although this information is essential to design rational strategies for industrial strain improvement. In this study, we applied comparative genomics to identify all genetic changes occurring during the development of an erythromycin overproducer obtained using the traditional mutate-and- screen method.

**Results:**

Compared with the parental *Saccharopolyspora erythraea *NRRL 2338, the genome of the overproducing strain presents 117 deletion, 78 insertion and 12 transposition sites, with 71 insertion/deletion sites mapping within coding sequences (CDSs) and generating frame-shift mutations. Single nucleotide variations are present in 144 CDSs. Overall, the genomic variations affect 227 proteins of the overproducing strain and a considerable number of mutations alter genes of key enzymes in the central carbon and nitrogen metabolism and in the biosynthesis of secondary metabolites, resulting in the redirection of common precursors toward erythromycin biosynthesis. Interestingly, several mutations inactivate genes coding for proteins that play fundamental roles in basic transcription and translation machineries including the transcription anti-termination factor NusB and the transcription elongation factor Efp. These mutations, along with those affecting genes coding for pleiotropic or pathway-specific regulators, affect global expression profile as demonstrated by a comparative analysis of the parental and overproducer expression profiles. Genomic data, finally, suggest that the mutate-and-screen process might have been accelerated by mutations in DNA repair genes.

**Conclusions:**

This study helps to clarify the mechanisms underlying antibiotic overproduction providing valuable information about new possible molecular targets for rationale strain improvement.

## Background

Actinomycetes are ecologically important microorganisms that hold a prominent position in the industry due to their ability to produce a wide range of secondary metabolites with biological activities including antibiotics, anti-tumour agents and immuno-suppressants [1]. However, these microorganisms must often be genetically improved for higher production before they can be used in an industrial setting. Historically, strain improvement has been empirically carried out by multiple rounds of random mutagenesis and screening [2]. Since the late 1970s, the availability of molecular genetics tools and information about the biosynthetic pathways and genetic control for most of secondary metabolites of commercial interest has opened the way for improving strains through engineering-based approaches [3,4]. More recently, these rational strain improvement strategies benefit from the support of genomic, transcriptomic, proteomic, and metabolomic technologies [5-12]. Combining classical and recombinant strain improvement with a solid fermentation development program represents the optimal synergy to design commercially successful processes.

The erythromycin fermentation has been improved by the traditional mutate-and-screen method over the past 50 years. Erythromycin biosynthesis in the mycelial actinomycete, *Saccharopolyspora erythraea*, has been widely studied as a model system for antibiotic production [13-16] and erythromycin and its semi-synthetic derivatives are widely used in the clinic. As such, the development of improved producers still represents a challenging and up- to-date issue. Erythromycin A is obtained through a three-stage pathway [17], i.e., i) assembly of the 14-membered macrolactone 6-deoxyerythronolide B (6DEB) from one propionyl-CoA and six (2*S*)-methylmalonyl-CoA units by multifunctional modular polyketide synthase followed by ii) its hydroxylation to erythronolide B (EB), formation of the deoxysugars mycarose and desosamine from glucose and their addition to EB to make erythromycin D, and then iii) C-12 hydroxylation and C-3" O-methylation of the latter compound to produce erythromycin A [18,19].

Extensive genetic studies have provided some insight into the genes involved in erythromycin biosynthesis [20,21]. The erythromycin gene cluster contains 20 genes arranged in four major polycistronic units [22]. Evidence for regulatory genes has been missing for a long time hampering efforts to enhance erythromycin production other than by medium manipulation, random mutagenesis and selection. In recent times, the availability of the entire genome sequence of *S. erythraea *and the advent of metabolic engineering opened the possibility to deeply investigate the molecular mechanisms controlling erythromycin production [23-25]. Whole-genome approaches led, for instance, to the identification of BldD, a key developmental regulator in actinomycetes [26,27], as one of the major regulators of erythromycin synthesis [28]. At the same time, metabolic engineering evidenced that, manipulating the methylmalonyl-CoA metabolite node in *S. erythraea *and in *Aeromicrobium erythreum*, a non-filamentous erythromycin A producer [29,30], i.e., increasing the flux through feeder metabolic pathways, strongly influences the erythromycin yields.

Lately, new global approaches based on "RNA polymerase and ribosome engineering" have been successful used to improve erythromycin production under laboratory conditions. It has been shown that several mutations affecting *rpsL *(coding for the ribosomal protein S12) result in a marked enhancement of erythromycin production, accompanied by increased transcription of *bldD *[31]. It has been reported also that several mutations in *rpoB *(coding for the beta subunit of RNA polymerase) deeply change the transcriptional profile of *S*. *erythraea*. In particular, the expression of genes coding for key enzymes of carbon (and energy) and nitrogen central metabolism is dramatically altered affecting in turn the flux of metabolites through erythromycin feeder pathways [32]. Mutations in ribosomal protein- and RNA polymerase subunit-encoding genes can be easily selected in the presence of drugs opening the way for a new approach to strain improvement. Very recently, complete biosynthesis of erythromycin A and designed analogs has been obtained using *E. coli *as a heterologous host suggesting alternative strategies to improve erythromycin production [33].

The focus of the present study is to investigate the molecular mechanisms leading to erythromycin over-production in a classically improved strain by using a genomic approach. To this purpose, we identified all genetic changes that occurred in *S. erythraea *NRRL 2338/Px (hereafter indicated as *S. erythraea *Px), an erythromycin overproducer obtained through the traditional mutate-and-screen method. Compared with parental *S. erythraea *NRRL 2338, a total of 117 deletion sites, 78 insertion sites and 12 transposition sites were found across the genome of the overproducer. Moreover, single nucleotide variations affecting a total of 144 CDSs were identified between the two genomes. All genetic changes have been carefully mapped in *S. erythraea *Px genome and genomic information has been used to elucidate the molecular mechanism underlying the overproduction of erythromycin by this strain. Genomic comparison has been supported by comparative transcriptome, an approach that has been also successful used before this study [34], and phenotypic analysis.

## Results and discussion

### Genomic comparison of *S. Erythraea *px and *S. Erythraea *NRRL 2338

**Figure 1:**
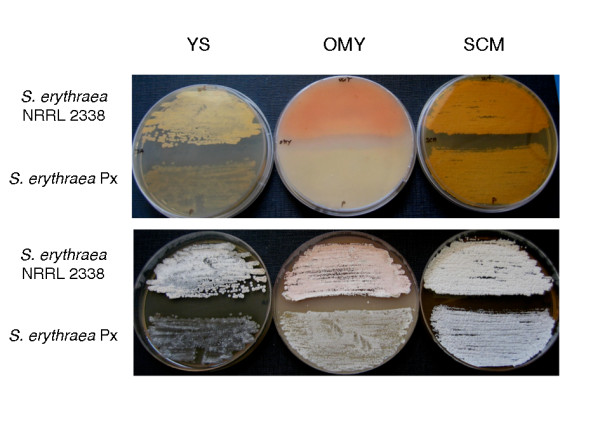
**Phenotypes of *S. erythraea *NRRL 2338 and *S. erythraea *Px cultivated on agar media**. Strains were cultivated for 7 days on YS, OMY or SCM agar plates as shown. Bottom (upper panel) and top (lower panel) sides of the agar plates were photographed.

Phenotypic differences between *S. erythraea *Px and the reference strain NRRL 2338 are shown in Figure 1. With respect to NRRL 2338, Px exhibited slower growth and reduced sporulation in both Yeast Starch (YS) and Oatmeal Yeast (OMY) agar plates, less pigmentation in OMY agar, while the phenotypes of the two strains were more similar in Soluble Complete Medium (SCM) agar. SCM broth was used in fermentation and microarray experiments (see below).

**Figure 2:**
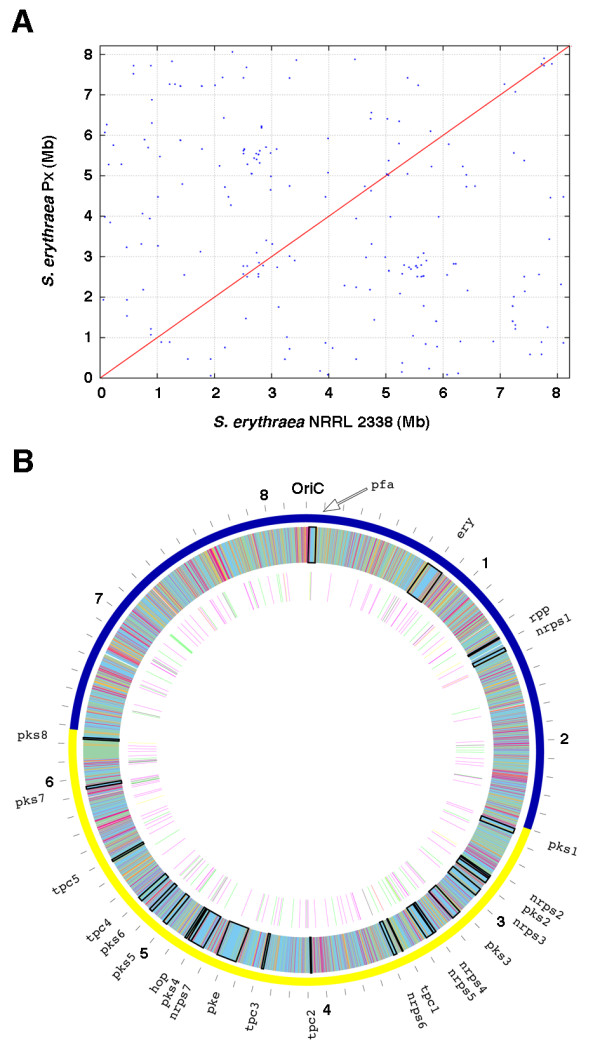
**Dot plot and chromosome map of genetic variations distinguishing *S. erythraea *Px from *S. erythraea *NRRL 2338**. A) Dot plot comparison beetwen the *S. erythraea *NRRL 2338 and Px strains generated by MUMmer software. B) Chromosome map of genetic variations distinguishing Px from NRRL 2338. The outer scale is numbered in megabases starting from the origin of replication (OriC), and indicates the core (blue) and noncore (yellow) regions. Outer circle: *S. erythraea *NRRL 2338 genescolor-coded by their COG function (orange, cellular process and signaling; purple, information storage and processing; light blue, metabolism; pale green, poorly characterized); inner circle: positions of variations between Px and NRRL 2338 color-coded by type (green, frameshift; purple, nonsense; yellow, missense). Position of secondary metabolism cluster genes are shown.

To understand the genetic bases underlying phenotypic differences between the two strains and improved erythromycin A production in Px, whole genome of this strain was sequenced and compared with that of NRRL 2338. The genome of Px consists of a single circular chromosome of 8,212,111 bp with an average G + C content of 71.14%. The size of the Px chromosome is 694 bp smaller than that of NRRL 2338. Genomic comparison revealed highly conserved gene content and gene order between these two strains (Figure 2A). The two genomes are 99.1% identical and there is no remarkable change in the chromosome structure as clearly shown in the dot-plot comparison.

Compared with NRRL 2338, a total of 117 deletion sites, 78 insertion sites and 12 transposition sites were found across the Px genome. Among them, 71 sites are single nucleotide insertions/deletions (indels), which affect coding sequences leading to frame-shift mutations, and only 11 are indels larger than 100 bp. The largest insertion is 1127 bp near position 5,393,000 which is located between an oxidoreductase (SACE_4827) and a transcriptional regulator (SACE_4828), while the largest deletion is 1147 bp in 6,849,979-6,851,124 (corresponding to SACE_6108, an ATP-dependent helicase) which is located between a methyltransferase (SACE_6107) and a glycosyl transferase (SACE_6109)

Apart from frame-shift mutations, single nucleotide variations (SNVs) affecting a total of 144 CDSs were identified between the two chromosomes. The SNVs generate 110 missense and 10 nonsense mutations. The variations, also including 4 CDS duplications, 7 transposon/transposable element insertions and 1 transposon excision, affect a total of 227 proteins in the Px genome. Chromosome map of genetic variations distinguishing Px from NRRL 2338 is shown in Figure 2B. Mutations are homogeneously scattered along the whole chromosome without "core genome" vs. "non-core genome" preference. The mutated CDSs, the corresponding gene/locus names, the nature of variations and presumed functions are reported in Tables 1, 2, 3, 4, 5, 6 with reference to the following aspects: i.) carbon, nitrogen and sulfur metabolism; ii.) substrate uptake, membrane permeability and secretion; iii.) biosynthesis of secondary metabolites; iv.) transcription and translation processes; v.) cell division, DNA replication and repair, transposition and phage integration; vi.) CDSs of unknown functions.

**Table 1:** Variations within genes related to central carbon, nitrogen and sulfur metabolism

Gene locus in NRRL 2338	Length (amino acids)	Gene/locus	Variation^a^	Protein function and notes
**Central carbon and energy metabolism**				

SACE_0618	286	*ccdA*	Missense (G38S)	Cytochrome c biogenesis protein

SACE_0633	221	*citA*	IPM	Citrate synthase (N-terminal)

SACE_1638	609	*sucB*	Missense (V574A)	2-oxoglutarate dehydrogenase complex E2 component (dihydrolipoamide succinyltransferase)

SACE_3073	375	*hypD*	Nonsense (Y30*)	Hydrogenase expression/formation protein HypD

SACE_5291	335	*dhaK*	Frameshift (-C 717)	Dihydroxyacetone kinase N-terminal containing protein (DhaK)

SACE_6118	1125	*pyc*	Frameshift (-C 1222)	Pyruvate carboxylase

SACE_6385	1206	*kgd (sucA)*	Missense (1773 S)	2-oxoglutarate dehydrogenase complex El component

SACE_6668	288	*sucD*	In frame insertion (-2081, -209V, -210M, -2111, -212G, -213E)	Succinyl-CoA synthetase, alpha subunit

**Nitrogen and amino acid metabolism**				

SACE_0635	122	*ureB*	Frameshift (-C 352)	Urease beta subunit

SACE_3800	689		Missense (P601L)	Assimilatory nitrate reductase catalytic subunit (selenocysteine-containing)

SACE_4319	426	*aspB*	Missense (Y185C)	Aspartate aminotransferase

SACE_5299	433		Missense (G103R)	D-amino acid deaminase

SACE_5427	417		Frameshift	D-amino acid dehydrogenase, small

SACE_5741	500	*gltD*	Frameshift (-G 1301)	Glutamate synthase NADH/NADPH, small subunit

SACE_6330	421		Frameshift (-G 324)	Fusion protein (ligase/carboxylase and argininosuccinate lyase)

SACE_6764	378		Missense (T47A) Frameshift (-C 436)	Alanine racemase

**Sulfur and amino acid metabolism**				

SACE_3346	517		Missense (C286R)	Sulfite oxidase/oxidoreductase, molybdopterin binding

SACE_4434	269	*tauC*	Missense (L221F)	Taurine transport system permease protein TauC

SACE_4651	279	*tauD*	Missense (G177S)	Taurine dioxygenase, 2-oxoglutarate-dependent

SACE_6133	158	*cdo2*	Missense (P151S)	Cysteine dioxygenase

**Sugar metabolism**				

SACE_0652	383	*iroB*	Missense (A325V)	Glycosyl transferase, related to UDP-glucuronosy transferase

SACE_1940	788	*lacZ2*	Missense (A769V)	Beta-galactosidase/beta-glucuronidase

SACE_3069	207	*gmhA*	Frameshift (-G 429)	Phosphoheptose isomerase

SACE_3071	223	*gmhA*	Frameshift (-C 75,-C 90, -C91, -T 95, -G 98, -G 99, -C 100, -G 126)	Phosphoheptose isomerase

SACE_3737	1001	*ama2*	Missenses (R121A,N122Q, T123A, F124L, I125H, V127R)	Alpha-mannosidase

SACE_4596	814		Missense (L810Q)	Beta-galactosidase/beta-glucuronidase

SACE_5147	1099	*embC*	Nonsense (S399*)	Arabinosyl transferase

SACE_5208	1098	*embC*	Missense (S799F)	Arabinosyl transferase

SACE_5734	657		Frameshift (+A 1027/1028)	Galactofuranosyl transferase

SACE_6416	201	*rmlC*	Missense (A173T)	dTDP-4-dehydrorhamnose 3,5-epimerase

SACE_6564	328	*deoC*	Missense (G16S)	Deoxyribose-phosphate aldolase

SACE_6765	501		Missense (R207G, P208R)	Ribokinase

SACE_6971	433		Missense (S23F)	Glycosyl transferase

**Fatty acid metabolism**				

SACE_0351	281	*estC*	Missense (A211T)	Esterase

SACE_3132	275	*abmC*	Missense (V246G) Frameshift (-C 742)	Enoyl-CoA hydratase/isomerase (rev hydr) Putative: 2-ketocyclohexanecarboxyl- CoA hydrolase (amino benzoate degradation)

SACE_3216	526		Frameshift (-G 1366)	Long-chain acyl-CoA synthetase

SACE_3361	329		Missense (T312I)	Glycerophosphoryl diester phosphodiesterase

SACE_3450	304		IPM	Enoyl-CoA hydratase/isomerase

SACE_3478	568		IPM	Long-chain acyl-CoA synthetase

SACE_3745	255		Missense (L187R)	Enoyl-CoA hydratase/isomerase

SACE_3936	246		Missense (L48H)	3-oxoacyl-[acyl-carrier protein] reductase

SACE_4589	388		Frameshift (+CACC 10/11)	Acyl-CoA dehydrogenase

**Nucleotide metabolism**				

SACE_1282	360	*nrdB*	Missense (S2N)	Ribonucleotide-diphosphate reductase subunit β

SACE_2080	432	*pyrC*	Missense (A90V)	Dihydroorotase

SACE_6664	521	*purH*	Missense (P54S)	Bifunctional phosphoribosylaminoimidazolecarboxa mide formyltransferase/IMP cyclohydrolase No paralogue.

SACE_7125	538	*purF*	Missense (P497S)	Glutamine phosphoribosylpyrophosphate amidotransferase No paralogue

SACE_2398	165	*prsA*	Missense (T123P)	Phosphoribosylpyrophosphate synthetase

SACE_5196	506	*codA*	Missense (Y322S)	Cytosine deaminase

**Vitamin and cofactor metabolism**				

SACE_0506	376	*thiO*	Missense (A44V)	Amino acid oxidase flavoprotein ThiO, putativeGlycine/D-amino acid oxidases

SACE_0511	547	*thiC*	Missense (T30I)	Thiamine biosynthesis protein ThiC

SACE_5955	1201	*cobN*	Missense (A1091T)	Cobaltochelatase subunit CobN

**Monooxygenases/oxidoreductases/methyltransferases/hydrolases of unknown function**				

SACE_0651	422	*cypA*	Frameshift (+C 16/17; +C 22/23)	Cytochrome P450

SACE_0781	191		Missense (P80S)	NADPH-dependent FMN reductase

SACE_4325	341		Frameshift (-C 890)	Radical SAM family protein Fe-S oxidoreductase

SACE_4560	326		Frameshift (+A 58/59)	SAM-dependent methyltransferase

SACE_4563	515		Missense (S9F)	Coproporphyrinogen III oxidase

SACE_4854	366		Frameshift (+G 477/478)	Amine oxidase, flavin-containing protein

SACE_5012	454		Missense (G114E)	Haem peroxidase

SACE_5030	372		Frameshift (-G 553)	Oye family NADH-dependent flavin oxidoreductase

SACE_5053	229		Missense (L204F)	Amidohydrolase 2

SACE_6588	491		Frameshift (-C 633)	Monooxygenase, FAD-binding

SACE_7243	462		Nonsense (W188*)	FAD-dependent oxygenase/FAD/FMN-containing dehydrogenase

aIPM, imperfect match

**Table 2:** Variations within genes related to substrate uptake, membrane permeability and secretion

Gene locus in NRRL 2338	Length (amino acids)	Gene/locus	Variation^a^	Protein function and notes
**Transport system**				

SACE_0429	646		Missense (P416L)	ABC-type transport system, permease component

SACE_0924	482		Frameshift (-C212;-C 221;-C 228; -G 264; -G 273)	Extracellular solute binding protein

SACE_0925	337		1PM	ABC-type transport system, permease component

SACE_0926	284		Missense (V18M, A89T)	ABC-type sugar transport system, permease component

SACE_0991	300		Frameshift (-G 370)	Dipeptide transport system permease

SACE_1587	297		Frameshift (-C 585)	Sugar transport system permease

SACE_1969	152		Missense (L90R)	Twin-arginine translocation pathway signal

SACE_2131	278		Missense (L275F)	Permease of the drug/metabolite transporter (DMT) superfamily

SACE_2701	276		Frameshift (-C 750)	ABC-type transport system, ATP binding component

SACE_3038	545		Frameshift (+C 1407/1408)	ABC-type transport system, ATP binding component

SACE_3524	489		Frameshift (+G 976/977)	Permease of the major facilitator superfamily

SACE_4034	426		Missense (L221P)	Permease of the major facilitator superfamily

SACE_4066	343		Frameshift (+C 268/269)	C4 dicarboxylate transporter/malic acid transport protein

SACE_4307	309		Missense (A90T)	Integral membrane transport protein

SACE_4347	555		Missense (A277G)	Sodium:solute symporter

SACE_4454	466		Missense (G348S)	Permease of the major facilitator superfamily

SACE_4982	407		Missense (L145F)	Integral membrane transport protein

SACE_5435	688		Missense and in frame deletion (N244T, G245A, A246P, D247D, R248A, L249A, H250R, G251R, G252L, L253T, Q255C, L256A, Q258H, A259R, T260G, L263-, P264-, T254S)	ABC-type transport system, permease component

SACE_5787	318		Missense (G300R)	Dipeptide ABC transporter, ATP-binding protein

SACE_6087	200	*kdpC*	IPM	Potassium-transporting ATPase, C chain

SACE_6246	229		Missense (R206C)	Phosphoenolpyruvate-protein phosphotransferase

SACE_6319	455		Frameshift (-C 1095)	Permease of the major facilitator superfamily

SACE_6323	315		Frameshift (-AT 913 and 914)	Dipeptide/oligopeptide/Nichel ABC transporter, permease component

SACE_6326	444		Frameshift (-G 1036)	Permease of the major facilitator superfamily

SACE_6578	364		Missense (G270R)	ABC-type sugar transport system, permease component

SACE_6927	392		Missense (P121L)	Permease of the drug/metabolite transporter (DMT) superfamily

SACE_6972	314		Missense (L224F)	Permease of the major facilitator superfamily

SACE_7047	403		Frameshift (+T 654/655)	Major facilitator superfamily sugar transporter

SACE_7202	327		Frameshift (+C 873/874)	Integral membrane transport protein

**Exo-enzymes**				

SACE_1076	326	*chiA2*	Nonsense (W315*)	Chitinase A

SACE_3366	364		Insertion of SACE-2828 (transposase)	Feruloyl esterase

SACE_3961	296		Missense (W21C)	Phospholipase D/diacylglycerol kinase

SACE_6961	263		Frameshift (-C 729)	Phospholipase D/diacylglycerol kinase

**Membrane and cell wall structures/enzymes**				

SACE_1186	202		Frameshift (+C 84/85)	Lipoprotein

SACE_2081	178		Missense (G62D)	Integral membrane protein

SACE_2338	338		Missense (P299R)	Membrane-bound lytic murein transglycosylase B

SACE_4420	192		Frameshift (-C 79)	Lipoprotein

SACE_5285	645		Frameshift (-T 29, +C 1890/1891)	Lipoprotein

^a^IPM, imperfect match

**Table 3:** Variations within genes related to biosynthesis of secondary metabolites

Gene locus in NRRL 2338	Length (amino acids)	Gene (locus)	Variation^a^	Protein function and notes
**Polyketide synthases and related biosynthetic proteins**				

SACE_0019	95	*(pfa)*	Nonsense (Q86*)	Acyl carrier protein

SACE_0022	752	*pfaB (pfa)*	Frameshift (+T 2003/2004)	Modular polyketide synthase *(pfa *gene cluster) (module 1: KR-ACP)

SACE_0023	2322	*pfaC (pfa)*	Missense (A317T,D1707N)	Modular polyketide synthase *(pfa *gene cluster) (module 2: KS-TE)

SACE_0718	237	*eryCVI (ery)*	Missense (T64A)	Erythromycin biosynthesis: TDP-desosamine-N-dimethy transferase

SACE_0720	322	*eryBIV (ery)*	Missense (G3E)	Erythromycin biosynthesis: dTDP-4-keto-6-deoxy-L-hexose 4-reductase

SACE_2595	2368		Missense (G1215S, D1118N)	Type I polyketide synthase (module: KS-AT-DH-ER-KR-ACP)

SACE_2630	4576	*pks2-l (pks2)*	Missense (A3004V)	Modular polyketide synthase (module 1: KS-AT(P)-ACP; module 2: KS-AT(A)-DH-KR-ACP; module 3: KS-AT(A)-DH-KR-ACP)

SACE_2875	4132	*(pks3)*	Frameshift (-G 2035)	Modular polyketide synthase *(pks3 *gene cluster (load: CL-ACP; module 1**: **KS-AT(P)-DH-ER-KR-ACP; module 2: KS-AT(A)-ACP)

SACE_2876	252	*gdmF (pks3)*	Missense (G17W)	3-amino-5-hydroxybenzoic acid synthase (AHBA synthase)

SACE_2888	455		Missense (V91C)	Aromatic-L-amino-acid decarboxylase

SACE_4140	3481	*pkeA2 (pke)*	Missense (F1282V)	Modular polyketide synthase (module 1: KS-AT(A)-DH-KR-ACP; module 2: KS-AT(A)-DH-KR-ACP)

SACE_5308	1730	*(pks7)*	Missense (A795T)	Iterative type 1 polyketide synthase

**Non-ribosomal peptide synthases and related biosynthetic proteins**				

SACE_3015	1083	*(nrps4)*	1PM	Non-ribosomal peptide synthase

SACE_3016	334	*(nrps4)*	Missense (V245F)	SyrP-like protein

SACE_3033	441		Missense (R186C)	Lysine/ornithine N-monooxygenase

SACE_3057	547		Missense (A481T)	2-polyprenyl-6-methoxyphenol hydroxylase

SACE_4288	7259	*(nrpsl)*	Missense (P6258L)	Non-ribosomal peptide synthase

**Terpene synthases and related biosynthetic proteins**				

SACE_3187	758	*(tpcl/geol)*	Nonsense (W83*)	Terpene synthase metal-binding domain-containing protein

SACE_3978	466	*(tpc3/geo2)*	Frameshift (+G 1040/1041)	Cyclic nucleotide-binding domain-containing protein *(tpc3 *gene cluster)

^a^IPM, imperfect match

Abbreviations: *CL *thioester ligase; *KSQ *Initiating decarboxylase; *KS *ketosynthase; *AT *acyltransferase (A and P refer to specificity for acetate and propionate units respectively); *ACP *acyl carrier protein; *KR *ketoreductase; *TE *thioesterase; *AMT *aminotransferase; *P450 *cytochrome P450 hydroxylase; *DH *dehydratase; *ER *enoylreductase

**Table 4:** Variations within genes related to transcription and translation processes

Gene locus in NRRL 2338	Length (amino acids)	Gene (locus)	Variation^a^	Protein function and notes
SACE_2076	148	*nusB*	Frameshift (-G 316)	NusB antiterminator factor No paralogue.

**Transcription factors**				

SACE 0891	206		Frameshift (+G 581/582)	TetR family transcriptional regulator

SACE_1040	163		Missense (R30C)	MarR family transcriptional regulator

SACE_1895	281		Frameshift (+C 603/604)	XRE family transcriptional regulator

SACE_2927	918		Missense (P52L)	SARP family transcriptional regulator

SACE_3079	294		Frameshift (-C 506)	LysR family transcriptional regulator

SACE_3348	96		Missense (E60A)	XRE family transcriptional regulator

SACE_4500	233		Frameshift (-C 129; -G 222)	GntR family transcriptional regulator

SACE_4536	327		Missense (T246N, L249M)	Lacl family transcription regulator

SACE_4775	246		Missense (A96V)	MerR family transcriptional regulator

SACE_5410	287		Missense (P140F)	XRE family transcriptional regulator

SACE_5425	505		Missense and in frame deletion (V297S, V298W, A299R, V300C, Q301R A302P, H303R, D304-, G305R)	PucR- family transcriptional regulator

SACE_5448- SACE_5449	126 150		Duplication of SACE_5448 and SACE_5449	SACE_5448: Unknown function SACE 5449: LuxR family transcriptional regulator

SACE_5739	282		Missense (K206T)	XRE family transcriptional regulator

SACE_6021	484		Frameshift (+A 1212/1213)	PucR- family transcriptional regulator

**Signal transduction**				

SACE_1833	241		Frameshift (-C 667)	Two-component system response regulator

SACE_1879	587		Frameshift (+C 1002/1003)	Serine/threonine protein kinase

SACE_1988	150		Missense (G125D)	Universal stress protein UspA

SACE_2583	541		Nonsense (R478*)	N-acyl D-amino acid deacylase

SACE_4937	414		Missense (G30R)	Mandelate racemase/starvation sensing protein

SACE_5284	286	*staP*	IPM	Endonuclease/Exonuclease/Phosphatase family protein

SACE_5286	224		Frameshift (-G419, -G 430)	Two-component system response regulator

SACE_5301	533		Frameshift (+C 1585/1586)	N-acyl_D-amino acid deacylase

SACE 6086	879	*kdpD*	Missense (P828L)	Osmosensitive K+ channel histidine kinase

SACE_6447	228	*mtrA*	In frame deletion (VI96-, HI97-)	Two-component system response regulator

SACE_6490	424		Missense (S37R)	Two-component system sensor kinase

SACE_6720	526	*phoD*	Missense (C328Y, P382F)	Phosphodiesterase/alkaline phosphatase D (phosphate starvation)

SACE_7263	382		Missense (A222T)	Two-component system sensor kinase

**Translation machinery**				

SACE_0443	464	*cysS*	Missense (G198S)	Cysteinyl-tRNA synthetase

SACE_0799	593	*metS*	Missense (P569S)	Methionyl-tRNA synthetase

SACE_2075	188	*efp*	Frameshift (+C 504)	Elongation factor P

SACE_3403	474	*gatA*	Missense (A243E)	Asp-tRNAAsn/Glu-tRNAGln amidotransferase A subunit

SACE_5926	1027	*infB*	Missense (A278V)	Translation initiation factor 2

SACE_5919	536		Frameshift (-G 821)	Pseudouridine synthase

**Protein turnover and chaperones**				

SACE_1339	860		IPM	Aminopeptidase N

SACE_2951	174	*clpC*	Frameshift (+G 468/469)	ATPases with chaperone activity, ATP-binding subunit

SACE_3756	768	*clpA*	Missense (I284T)	ATPases with chaperone activity, ATP-binding subunit

SACE_6784	610		Frameshift (-C 1573)	Molecular chaperone

SACE_6113	481		IPM?	Aminopeptidase

^a^IPM, imperfect match

**Table 5:** Variations within genes related to cell division, DNA replication and repair, transposition and phage integration

Gene locus in NRRL 2338	Length (amino acids)	Gene (locus)	Variation^a^	Protein function and notes
**DNA replication and repair**				

SACE_0826	1195	*mfd*	Frameshift (+A 218/219)	Transcription/repair coupling factor

SACE_1351	269	*mutM, fpg*	Missense (M199T)	Formamidopyrimidine DNA glycosylase

SACE_3677	147	*ogtl*	Missense (G41K)	Methylated DNA-protein cysteine methyltransferase

SACE_4427	183		Frameshift (+G 353/354)	G/U mismatch-specific DNA glycosidase

SACE_5437	895	*polA*	Frameshift (-C617)	DNA polymerase I

SACE_5255	207	*alkA*	Missense (P68F)	3-methyladenine DNA glycosylase

SACE_6108	693	*uvrD2*	IPM	ATP-dependent DNA helicase UvrD-like

			Missense and in	

SACE_6681	873	*uvrD*	frame deletion (G54-, G56R, S57I)	ATP-dependent DNA helicase UvrD

**Cell division**				

SACE_0667	941		Missense (F874L)	DNA segregation ATPase FtsK/SpoIIIE

SACE_6104	263		IPM	Cell division initiation protein

SACE_6929	579		Missense (P159R)	ATPase involved in chromosome partitioning

**Transposition and phage integration**				

SACE_0657	106		Excision	lS1647-like transposase

SACE_2154	350		IPM	Transposase, IS891/IS1136/IS1341

SACE_2214and similar CDSs	365		Insertion between SACE_4827 and SACE_4228	SACE-2214: Transposase SACE_4827: Oxidoreductase SACE_4228: LacI family transcriptional regulator

SACE_2313 and similar CDSs	99		Insertion between SACE_2371 and SACE_2372	SACE_2313: IS4-like transposase SACE_2371: IS4-like transposase SACE_2372: IS 1647-like transposase

SACE_2322	374		Missense (VI391)	Phage-related integrase/Site-specific recombinase XerC

SACE_3579	469	*tnp*	Missense (R13S, A73S, T118A, V309A)	Transposase, 1S1XX5

SACE_4072	469	*tnp*	Missense (S178L, Q191R, S199A,H218R, N370T, G430S, T431V)	Transposase, ISlxx5

SACE_5073	459		Missense (N171K)	Transposase, IS3508i

			Missense and in frame insertion	

SACE_5268	395		(S73T, A74P, H129N,-134T, -135V,L191V, I216V A296V, P388R, S396L)	Transposase inactivated by frameshift mutation)

SACE_5430	232		Missense (H15R, R193W)	Transposase, Tn5714

^a^IPM, imperfect match

**Table 6:** Variations within genes of unknown function

Gene locus in NRRL 2338	Length (amino acids)	Gene/locus	Variation^a^	Protein function and notes
**Unknown function**				

SACE_0062	35		Insertion betweenSACE_ 2351 and SACE_2352	SACE_0062: Unknown function. SACE_2351: Tn5714-like transposase. SACE_2352: IS 111 a/IS 1328/IS1533-like transposase.

SACE_0062	35		Insertion in SACE_5430, between SACE_5429 and SACE_5431	SACE_0062: Unknown function. SACE_5429: Dephospho-CoA kinase SACE_5430: Transposase, Tn5714 SACE_5431: 30S ribosomal protein SI

SACE_0157	389		Missense (P295L)	Unknown function/Glutathionylspermidine synthase

SACE_0587	995		Frameshift (+G 332/333)	Unknown function

SACE_0744	164		Missense (R42H)	Unknown function

SACE_0940	289		IPM	Unknown function

SACE_0944	831		Nonsense (Y412*)	Unknown function

SACE_1128	294		Missense (VI13 A)	Unknown function

SACE_1129	774		Missense (A172T)	Unknown function

SACE_1257	195		Nonsense (Q60*)	Unknown function/uncharacterized MobA-related protein

SACE_1344	1638		IPM?	Unknown function/PE-PGRS family protein

SACE_1805	100		Missense (S13F)	Unknown function

SACE_1835	175		IPM	Unknown function

SACE_1853	110		Frameshift (+G 51/52)	Unknown function

SACE_2384	496		Frameshift (+T 823/824)	Unknown function

SACE_2456	251		Missense (A28V)	Unknown function

SACE_2737	116		Missense (P4R)	Unknown function

SACE_3005	316		Frameshift (-G 727)	Unknown function

SACE_3102	1249		Frameshift (+G 338/339)	Unknown function

SACE_3186	392		Missense (R390S)	Unknown function *(tpcl *cluster)

SACE_3262	282		Frameshift (+C 434/435) Insertion between	Unknown function SACE 3264: Unknown secreted protein.

SACE_3264	117		SACE 3572 and SACE 3573	SACE 3572: Unknown secreted protein. SACE 3573: Unknown function

SACE_3748	277		Frameshift (+C 696/697)	Unknown function

SACE_3850	261		Frameshift (-G 388)	Unknown function

SACE_3925	188		Frameshift (+A 437/438)	Unknown function

SACE_4249	234		Frameshift (-G 640)	Unknown function

SACE_4310	480		Frameshift (-C 1146)	Unknown function

SACE_4451	291		In frame insertion (-102D,-103A, -104N,-105E, -106Q)	Unknown function

SACE_4989	38		Insertion between SACE_5103 and SACE_5105	SACE_4989: Unknown function. SACE_5103: Type III restriction enzyme, res subunit. SACE_5105: Transposase

SACE_5311	835		Missense (G411R) Frameshift	Unknown function

SACE_5423	184		(-G 372, -G 473, -C 481,-G 493,-T 409,-C 540)	Unknown function

SACE_5446	1184		Frameshift (-C 2898)	Unknown function

SACE_5460	330		Missense (P162F)	Unknown function

SACE_5482	632		Missense (L509P)	Unknown function

SACE_5483	11792		Missense (L1329F, T5002A, P8537L, R9518H)	Unknown function

SACE_5513	566		In frame deletion (S249-, E250-,G251-, T252-,T253-, G254-, G255-, T256-, G257-, G258-, A259-, G260-)	Unknown function

SACE_5523	5856		Nonsense (L370*)	Unknown function

SACE_5655	31		Duplication of SACE_5655	Unknown function

SACE_5905	418		Missense and in frame insertion (A5V, T7G, I8C, R10H, V11H, Q12L, T13A, M14G, S15A I16D, E17H, S18E, A19H, R21E, T22R, L24A, -25H, -26R, -27A)	Unknown function

SACE_6184	171		Frameshift (-G 169)	Unknown function

SACE_6567	206		Missense, in frame insertion, nonsense (A135G,-136R,- 137R,-138G,-139A,- 140A,-141R,-142W, -143G,-144P, -145P, -146R,-147S,-148*, A149R, L155R, G156A, T157R, A158-, A159-, V160-, V161-, T161-, F163-, A165*, A166R,I167T, A168G, T170-, V171-) V172-,K173-,D174-, W175-,F176-, V177-, A178R, A164S	Unknown function

SACE_6773	962		Missense (D330E) Frameshift (-G 1654)	Unknown function/PE-PGRS family protein

SACE_7193	278		Frameshift (+C 729/730)	Unknown function

SACE_7240	265		Missense (P90H)	Unknown function

SACE_7316	47		Duplication of SACE_7316	Unknown function

^a^IPM, imperfect match

### Transcriptome comparison of *S. Erythraea *px and *S. Erythraea *NRRL 2338

**Figure 3:**
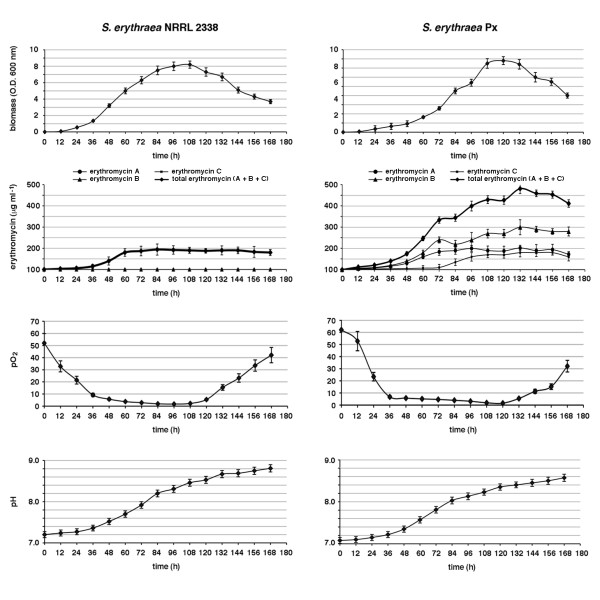
**Bioreactor cultures of *S. erythraea *NRRL2338 and *S. erythraea *Px**. Biomass, erythromycin production, pO2 and pH were evaluated as described in the Materials and Methods section. Erythromycin production was monitored by HPLC. Total erythromycin is the sum of erythromycin A, B and C. Values are means ± S.D. of three independent experiments for each strain starting from the same frozen culture.

To gain further insight about the molecular mechanisms underlying improved erythromycin A production in Px strain, DNA microarray of *S. erythraea *were manufactured and used for comparative analysis between Px and NRRL 2338. Microarray data for NRRL 2338 strains were already available [24]. In DNA microarray experiments Px and NRRL were cultivated under standard batch-culture conditions in SCM broth [29] and erythromycin production was evaluated at different time points (Figure 3).

Due to the different length of lag phase, RNA samples were collected during growth of NRRL 2338 in the time interval 12-72 h, and in the time interval 24-84 for Px. Despite similar final values of biomass, shapes of growth and erythromycin production curves were markedly different between the two strains. Erythromycin production by both strains was detectable after 12 h. Consistently with previous findings [24], three distinct phases could be distinguished during erythromycin fermentation in the reference strain: an initial period of rapid increase of antibiotic concentration lasting until 60 h (phase **a**), followed by a period of production slowdown until 72 h (phase **b**), and a second period of moderate increase of erythromycin titers from 72 to 84 h (phase **c**) before entering the stationary phase. In the erythromycin overproducer phase **a **was protracted longer up to 72 h, phase **b **lasted until 84 h, while phase **c **was characterized by gradual increase of erythromycin titers until 108 h with a further period of production slowdown between 108 and 120 h followed by a further increase up to 132. Therefore, while during growth of NRRL 2338 erythromycin titers remained stable over the stationary phase, during growth of Px the antibiotic concentration continued to increase in the exhausted medium after entering the stationary phase reaching final titers about five-fold higher than in NRRL 2338. Moreover, while NRRL 2338 produced essentially erythromycin A during fermentation, Px yielded erythromycin A along with significant amounts of its immediate precursors erythromycin B and erythromycin C (Figure 3).

**Figure 4:**
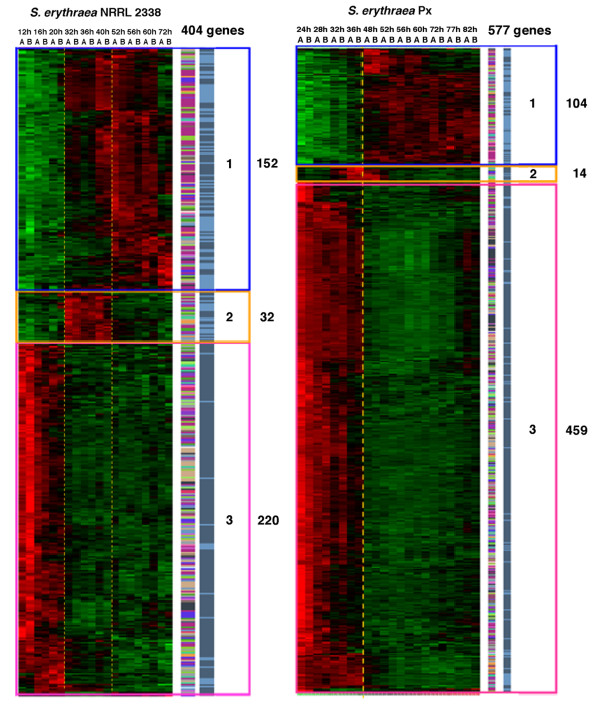
**Microarray analysis showing changes in global control of cell-cycle**. Visualization by dChip of 404 *S. erythraea *NRRL2338 and of 577 *S. erythraea *Px differentially expressed genes, selected by a q-value < = 0,001, and determining a time course gene expression profiling and a hierarchical clustering of the samples (Red = up-regulation; Green = down-regulation); on the right side of the figure, two columns respectively show the functional classification of the DEGs whose transcription profile is visualized and their positional distribution in the core (dark blue) and non core region (light blue) of the genome.

Gene expression data were analyzed to identify transcripts modulated during the growth curve. Considering each time point replicate as an independent entry and setting the confidence threshold at q-value ≤ 0.001 (see Methods), the EDGE algorithm identified a total of 404 differentially expressed genes (DEGs) in NRRL 2338 (6.22% of total probeset) with 220, 32 and 152 genes up-regulated, respectively, during phase **a**, **b **and **c**. In the Px strain the number of DEGs identified increased to 577 (8.88% of total probeset) and only two clusters of genes up-regulated, respectively during phases **a **(459 genes) and phase **b **(104 genes) could be clearly distinguished. Microarray analysis confirmed changes in global control of cell cycle in Px strain with respect to NRRL 2338 (Figure 4).

Inspection of functional classification of cell cycle-regulated genes showed that the increment in the number of DEGs in Px was mostly due to genes belonging to the following clusters of orthologous groups (COGs): I.5 Intracellular trafficking, secretion, and vescicular transport; I.6 Posttranslational modification, protein turnover, chaperones (in the functional category Cellular processes and signaling); II.12 Translation, ribosomal structure and biogenesis; III.8 Nucleotide transport and metabolism; III.9 Posttranslational modification, protein turnover, chaperones (in the functional category Metabolism) (Table 7). At the same time the following clusters of orthologous group resulted under-represented: II.11 Transcription and III.3 Cell wall/membrane/envelope biogenesis. In Table 7 the COGs have been considered over- represented (bold numbers in Table 7) if the percentage of the genes belonging to each category respect to the total of DEGs resulted 2 times more than the percentage of the genes belonging to each category respect to the total of GeneChip probesets. On the contrary they were considered under-represented (oblique numbers in Table 7) if the percentage of the genes belonging to each category respect to the total of DEGs resulted less than half of the percentage of the genes belonging to each category respect to the total probesets. A complete list of the cell cycle-regulated genes in NRRL 2338 and Px is available as supplemental data files (Additional files 1, 2), while the lists of genes up-regulated during phase **a **either in both strains or in Px or in NRRL 2338 are reported, respectively, in Additional files 3, 4, 5.

**Table 7:** Functional classification of cell cycle-regulated genes in both strains^a^

		Strain	NRRL2338	Px
**COG**	**Functional categories**	**% COG probesets**	**% Probesets with q-value < = 0.001**	**% Probesets with q-value < = 0.001**

				

**I**	**Cellular processes and signaling**			

I.1	Cell cycle control, cell division, chromosome partitioning	0,35	**0,99**	0,69

I.2	Cell motility	0,01	0	0

I.3	Cell wall/membrane/envelope biogenesis	2,05	3,22	3,81

I.4	Defense mechanisms	0,89	0,00	0,87

I.5	Intracellular trafficking, secretion, and vesicular transport	0,24	0,25	**0,69**

I.6	Posttranslational modification, protein turnover, chaperones	1,90	**5,94**	**3,99**

I.7	Signal transduction mechanisms	1,91	0,99	1,91

**II**	**Information storage and processing**			

II.1	Amino acid transport and metabolism	0,01	0	0

II.2	Cell wall/membrane/envelope biogenesis	0,01	0	0

II.3	Chromatin structure and dynamics	0,01	0	0

II.4	General function prediction only	0,30	0,50	0,35

II.5	Nucleotide transport and metabolism	0,07	0	0

II.6	Posttranslational modification, protein turnover, chaperone	0,04	0	0

II.7	Replication, recombination and repair	2,44	*0,74*	2,43

II.8	RNA processing and modification	0,01	0	

II.9	Secondary metabolites biosynthesis, transport catabolism	0,04	0	

II.10	Signal transduction mechanisms	0,71	0,50	0,69

II.11	Transcription	7,20	*2,23*	*3,29*

II.12	Translation, ribosomal structure and biogenesis	2,48	4,21	**10,75**

**III**	**Metabolism**			

III.l	Amino acid transport and metabolism	6,60	0	8,15

III.2	Carbohydrate transport and metabolism	6,08	7,67	6,59

III.3	Cell wall/membrane/envelope biogenesis	0,38	0	*0,17*

III.4	Coenzyme transport and metabolism	2,63	5,20	4,33

III.5	Energy production and conversion	4,79	**9,90**	**8,15**

III.6	Inorganic ion transport and metabolism	2,68	4,21	2,25

III.7	Lipid transport and metabolism	4,24	2,72	5,03

III.8	Nucleotide transport and metabolism	1,27	**2,97**	**3,64**

III.9	Posttranslational modification, protein turnover, chaperones	0,08	0	**0,17**

III.10	Secondary metabolites biosynthesis, transport catabolism	2,90	2,48	2,77

III. 11	Signal transduction mechanisms	0,16	0	0

**IV**	**Poorly characterized**			

IV.1	Function unknown	32,11	27,23	22,70

IV.2	General function prediction only	7,37	8,17	6,59

	Total probesets	7060	404	577

aThe categories over-represented and under-represented are evidenced, respectively, by **BOLD **and *oblique *number

**Figure 5:**
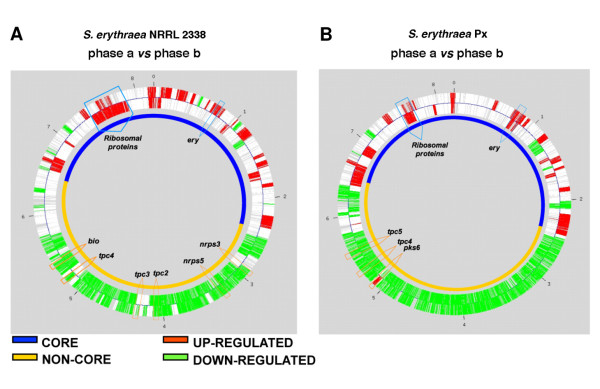
**Visualization of the LAP algorithm results on the two chromosome strands independently analyzed**. Comparison between the transcriptional profiling of all the 6494 *S. erythraea *genes in phase A versus phase B in the NRRL2338 strain(A) and in the Px strain(B) is shown. A q value of 0,01 and a fold change of 0,5 were chosen as filtering parameters. The transcriptionally up-modulated regions are shown in RED while the down- modulated are in GREEN. The OriC is indicated as O and the resolution of the chromosome is 1 Mb. The core region is evidenced in blue and the non-core region in orange; the position of clusters involved in the secondary metabolism, erythromycin production and coding for ribosomal proteins are outlined by arrows.

The Locally Adaptive Statistical Procedure (LAP) [35] of the PREDA package [ref] was used to identify differentially expressed chromosomal regions (Figure 5). LAP analysis showed significant up-regulation of many genes clustered in the "core" region of the chromosome (mostly containing essential genes) in both NRRL 2338 and Px strains during phase **a**, including the erythromycin biosynthetic cluster (*ery*) and the ribosomal proteins-encoding operons (Figure 5A and 5B). In contrast most of genes clustering in the "non-core" region of the chromosome (mostly containing "contingency" genes) were down-regulated during this growth phase. The only notable exception was represented by *pks6 *cluster coding for an unknown type II polyketide [23], which was up-regulated in Px strain during phase **a**. Moreover, while in NRRL 2338 the biosynthetic clusters *nrps3*, *nrps5*, *tpc2*, *tpc3 *and *tpc4 *clearly appeared to be down-regulated during phase **a**, in Px only two clusters, *tpc4 *and *tpc5*, exhibited this behavior suggesting profound differences in control of secondary metabolism between the two strains (Figure 5A and 5B). The LAP analysis failed to identify regions exhibiting significant differential gene expression between phase **b **and phase **c **in NRRL 2338 see Peano et al. 2007 [24].

### Central carbon and nitrogen metabolism in *S. Erythraea *px

Table 1 shows that a considerable number of mutated genes are involved in central carbon (and energy) and nitrogen metabolism, or related to substrate uptake and utilization (Table 2). This is not surprising because erythromycin biosynthesis is strictly connected with central metabolism consistently with both genomic and expression data. In general, secondary metabolism is believed dispensable for survival, and most of gene clusters coding for secondary metabolites occupy non-core genomic regions and are maximally expressed during late growth phases. In contrast, the *ery *cluster maps in the core region of the *S. erythraea *chromosome, and is transcribed during the middle pseudo-exponential growth phase when the activities if the carbon and nitrogen central metabolic pathways are maximal (Figures 4 and 5) [23,24,32,36].

**Figure 6:**
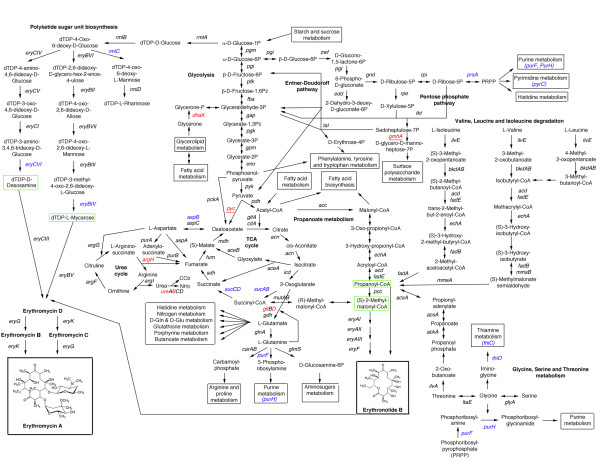
**Overview of carbon and nitrogen metabolic pathways and their relationships with the biosynthesis of erythromycin**. Genes affected by missense mutations are indicated in blue; genes affected by missense/nonsense mutations are shown in red. Single-copy genes are underlined.

As shown in Figure 6, these pathways are strictly connected to erythromycin biosynthesis that requires one propionyl-CoA and six (2S)-methylmalonyl-CoA units for assembly of the 14- membered macrolactone 6DEB. Different metabolic routes may accomplish precursor supply for erythromycin biosynthesis. In addition to well-established propanoate metabolism leading to both propionyl-CoA and six (2S)-methylmalonyl-CoA units, alternative routes exist including valine, leucine and isoleucine degradation pathways, and glycine, serine and threonine metabolism. An additional proposed route to (2S)-methylmalonyl-CoA proceeds by the rearrangement of succinyl-CoA catalyzed by methylmalonyl-CoA mutase yielding the (2R)- isomer of methylmalonyl-CoA that would be converted to the (2S)- isomer by a methylmalonyl-CoA epimerase.

Our analysis demonstrated that the several genes coding for the component enzymes of the tricarboxylic acid cycle (TCA) were mutated in Px strain. In particular, *pyc *gene (SACE_6118) coding for pyruvate carboxylase (PC) was inactivated by a frameshift mutation. This result is noteworthy, because PC serves a major anaplerotic role for the TCA by catalyzing the ATP-dependent carboxylation of pyruvate to oxalacetate, and there is evidence that in *Corynebacterium glutamicum *PC contributes about 90% to C(3) carboxylation at the anaplerotic node (Petersen, 2000). Moreover, *pyc *has no obvious paralog(s) in the genome of *S. erythraea*. In addition to *pyc*, non-conservative missense mutations affected both the E1 and the E2 component-encoding genes (*sucA *[SACE_6385] and *sucB *[SACE_1638], respectively) of the 2-oxoglutarate dehydrogenase complex. 2- oxoglutarate dehydrogenase is a key enzyme in the TCA cycle, converting 2-oxoglutarate, coenzyme A and NAD(+) to succinyl-CoA, NADH and carbon dioxide. This activity is tightly regulated and it is a major determinant of the metabolic flux through the TCA cycle. Also in this case, obvious paralogs could not be found in the genome of this microorganism. Although it is difficult to predict the effect of the observed mutations on the activity of the 2- oxoglutarate dehydrogenase complex, altogether our results are consistent with the hypothesis that the carbon flux through the TCA cycle may be greatly reduced in the Px strain, and that, as a consequence, more acetyl-CoA is funneled into alternative routes including propanoate metabolism leading to the 6DEB precursors (Figure 6).

**Figure 7:**
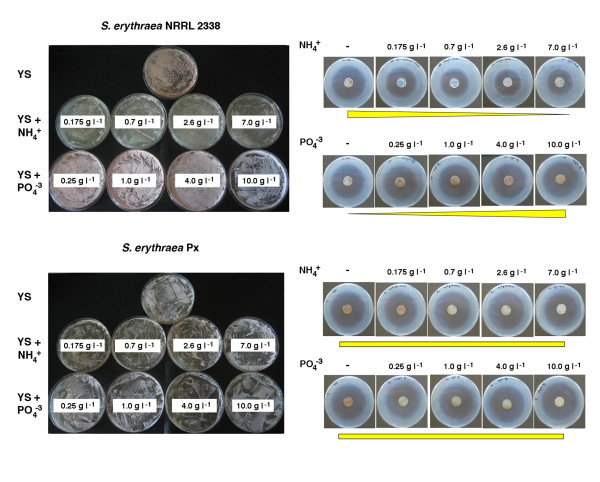
**Effect of ammonia and phosphate on growth, pigmentation, sporulation and antibiotic production**. *S. erythraea *NRRL 2338 and *S. erythraea *Px were cultivated for 7 days on YS agar supplemented with the indicated ammonia and phosphate concentrations (left panels). Antibiotic production in these media was evaluated by microbiological assay using *Micrococcus luteus *as a tester organism (right panel)

This view is supported by evidence that glutamine synthatase/glutamate synthase (GS- GOGAT) and urea cycles, which are linked to TCA by 2-oxoglutarate and oxaloacetate/fumarate, respectively, may also be affected in the Px strain. Indeed, in this strain, *gltD*, coding for small subunit of glutamate synthase (SACE_5741), a component of the high-affinity ammonium assimilation system, is inactivated by a frameshift mutation (Table 1 and Figure 6). However, in this case, a paralogous gene (SACE_3997) is predicted to exist in the genome, to alleviate the deleterious effect of such a mutation. A possible deficiency in the high-affinity ammonium assimilation system may also account for the different behavior of *S. erythraea *NRRL 2338 and Px strain with respect to ammonium content in the growth medium. Growth, pigment and antibiotic production was strongly inhibited in the wild type strain in the presence of high ammonium content, while, in contrast, the same parameters were not affected by ammonium in the erythromycin over-producing strain (Figure 7). Frameshift mutations inactivate SACE_6330 (*argH *domain) and SACE_0635 (*ureB*). These CDSs may be involved in the urea cycle as they code, respectively, for a fusion protein with both ligase/carboxylase and argininosuccinate lyase domains, and urease beta subunit. In the latter case, no obvious *ureB *paralog could be detected in the genome. In addition to the urea cycle, one of the two aspartate aminotransferase-encoding paralog (*aspB*) (SACE_4319), whose activity is linked to the urea cycle and is essential for growth on L-glutamate, was also affected by a non-conservative missense mutation.

In the absence of sulfate and cysteine, several bacteria can use aliphatic sulfonates as a source of sulfur for growth. *tauABCD *genes coding for an ABC-type transport system required for uptake of aliphatic sulfonates and a desulfonation enzyme accomplish this property. In Px strain *tauC *(SACE_4434) coding for permease component of taurine transport system, and *tauD *(SACE_4651) coding for 2-oxoglutarate-dependent taurine dioxygenase are affected by missense mutations, along with *cdo2 *(SACE_6133) encoding a cysteine dioxygenase, which catalyzes the conversion of L-cysteine to cysteine sulfinic acid, a compound that lies at a branch-point in cysteine catabolism, where it can follow two pathway resulting in the formation of taurine or sulfate (Table 1). Down-modulation or inactivation of this metabolic pathway involving the 2-oxoglutarate-dependent taurine dioxygenase may be functional to alleviate the reduced carbon flux through the Krebs cycle by increasing the pool of 2- oxoglutarate.

Several genes involved in nucleotide and thiamine metabolism were also affected by non- conservative missense mutations in the Px strain, including *prsA *(SACE_2398) coding for phosphoribosylpyrophosphate (PRPP) synthase (which catalyzes the synthesis of the common precursor for biosynthesis of histidine and purine and pyrimidine nucleotides), *purF *(SACE_7125) and *purH *(SACE_6664) whose products code for glutamine PRPP amidotransferase and bifunctional phosphoribosylaminoimidazolecarboxamide formyltransferase/IMP cylohydrolase (which catalyzes, respectively, the first and the second steps in the *de novo *biosynthesis of thiamine and purine nucleotides), *thiO *(SACE_0506) and *thiC *(SACE_0511) specifically involved in thiamine biosynthesis, and *pyrC *(SACE_2080) encoding dihydroorotase, a key enzyme in pyrimidine nucleotide biosynthesis (Table 1 and Figure 6). Mutations in the above-mentioned genes are expected to reduce the carbon flux toward these biosynthetic pathways (purine/thiamine, pyrimidine), and increase the flux toward the pentose phosphate (*prsA *mutation) and glycine, serine and threonine metabolic pathway (*purF*, *purH*, *thiO*, *thiC *mutations). The last pathway is a source of precursors for 6DEB biosynthesis.

The above-mentioned genetic defects in metabolic genes are well correlated with the slow- growth phenotype of the Px strain in mineral medium MM-101, caused by nutritional requirements which are indicative of TCA cycle precursors and PRPP limitations (Additional file 6). At the same time, our results emphasize the balance that must be reached between pathways competing for the same substrate to maintain robustness of the metabolic network.

### Secondary metabolism in *S. Erythraea *px

In addition to the gene clusters for erythromycin (*ery*), for a second modular polyketide synthase (PKS) of unknown function (*pke*) and for a type III PKS (*rppA*), which generates a reddish pigment, the genome sequencing of *S. erythraea *NRRL 2338 has revealed additional 22 clusters for the biosynthesis of polyketides, terpenes and non-ribosomally synthesized peptides. In Px strain a total of 19 missense/nonsense/frameshift mutations affected genes related to biosynthesis of secondary metabolites (Table 3).

Twelve of them affected genes coding for PKS or related biosynthetic proteins. In particular, a nonsense mutation in SACE_0019 (coding for an ACP), a frameshift mutation in SACE_0022 (*pfaB*) (coding for a modular PKS) and two non-conservative missense mutations in SACE_0023 (*pfaC*) (coding for a modular PKS) inactivated the *pfa *cluster, which appears to govern the biosynthesis of a polyunsaturated fatty acid such as eicosapentaenoic acid. The *pks3 *cluster was inactivated by a frameshift mutation in SACE_2875 (coding for a modular PKS) and a non-conservative missense mutations in SACE_2876 (*gdmF*) (coding for 3-amino-5-hydroxybenzoic acid synthase). Missense mutations affected the *pks3*-associated SACE_2888 (coding for an aromatic-L-amino acid decarboxylase). Non-conservative missense mutations were mapped in SACE_2630 (*pks2-1*) (coding for a modular PKS) of the biosynthetic cluster *pks2*, SACE_4140 (*pkeA2*) (coding for a modular PKS) in the *pke *cluster, and SACE_5308 coding for a multifunctional single- module PKS enzymes apparently related to the iterative PKSs involved in enediyne or methylsalicylic acid synthesis. It is conceivable that some of these pathways may compete with that of erythromycin for the same substrates and that their inactivation/down-modulation may be beneficial to 6DEB biosynthesis.

While it is difficult to predict the effects of the missense mutations affecting *eryCVI *and *eryBVI *(Table 3) coding for the enzymes catalyzing the last steps of the biosynthesis of dTDP-D-desosamine and dTDP-L-mycarose biosynthesis, the activated sugars that decorate the erythronolide B, the missense mutation affecting SACE_6416 (*rmlC*) coding for dTDP-4- dehydrorhamnose 3,5-epimerase (Table 1 and Figure 6) may cause an increase in production of both dTDP-D-desosamine and dTDP-L-mycarose precursors. In fact, the *rmlC*, *eryCIV *and *eryBVI *gene products compete for the same substrate (dTDP-4-oxo-6-deoxy-D-glucose). Additional work is required to clarify these aspects, as well as the possible effects of the inactivation/down-modulation of *nrps4 *and *nrps7 *biosynthetic clusters, coding for unknown non-ribosomally synthesized peptides, and of *tpc1/geo1 *and *tpc3/geo2 *showing substantial similarity to terpene cyclase-encoding clusters, which in other microorganisms are known to produce geosmin, the sesquiterpene that provide the soil with its characteristic smell.

### Global and pathway-specific control of gene expression, and DNA repair mechanisms in *S. Erythraea *px

**Figure 8:**
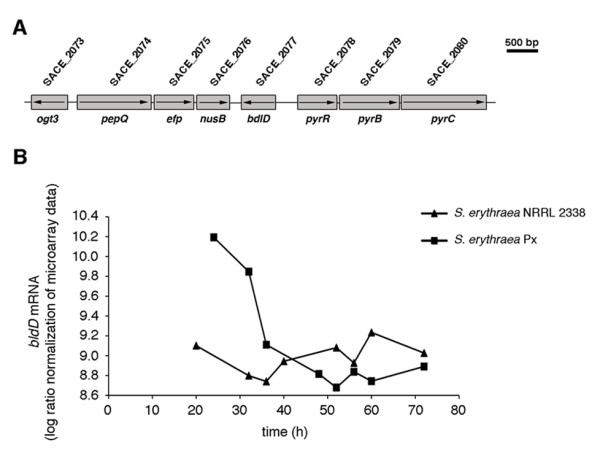
**Expression of *bldD *during growth of *S. erythraea *NRRL 2338 and *S. erythraea *Px**. A) Genetic map of the chromosomal region spanning the *pepQ-efp*-*nusB *and *bldD *genes. B) *bldD *mRNA levels are expressed as average of log ratio normalized microarray duplicate data (RMA). *bldD *expression values from *S. erythraea *NRRL 2338 and *S. erythraea *Px do not vary more than 1.58% and 2.61%, respectively, on the average.

Antibiotic production is under global and local pathway-specific control. At the same time, there is evidence that genetic manipulation of RNA polymerase and/or ribosome may influence the control of secondary metabolism [31,32]. Mutations affecting the basic transcription and translation machineries were thus expected in the erythromycin- overproducing strain (Table 4). However, unexpectedly, several inactivating mutations mapped in a number of genes, whose products were thought to play fundamental roles in these processes including *nusB *(SACE_2076) coding for NusB antiterminator factor and *efp *(SACE_2075) encoding the elongation factor P (EF-P). Both these genes, which are organized in a putative conserved operon also including *pepQ *gene coding for Xaa-Pro dipeptidase, were inactivated by frameshift mutations (Figure 8A).

NusB participates together with NusE/S10 protein in processive transcription antitermination. NusB and NusE, bind to form a heterodimer, which interacts with a specific boxA site on the RNA. The NusB/NusE/boxA RNA ternary complex interacts with the RNA polymerase transcription complex, stabilizing it and allowing transcription past premature termination points [37-40]. NusB is essential to suppress transcription termination in the ribosomal RNA (*rrn*) operons. Compared to wild type *Escherichia coli*, about two-fold decreased RNA polymerase density was observed by electron microscopy over 23S genes in a *nusB *mutant [41]. As a consequence, the fraction of total RNA polymerase engaged in transcribing the *rrn *operons is significantly reduced in a *nusB *mutant. Similar reduction is observed during the stringent response, when (p)ppGpp binds RNA polymerase and changes the global transcriptional profile, decreasing the synthesis of translational machinery and increasing the transcription of biosynthetic genes including those coding for antibiotics in actinomycetes [32,42-48].

EF-P is a highly conserved protein that is essential for protein synthesis in several bacteria including *E. coli *[49]. It has been suggested that EF-P plays a role in translational fidelity, prevents entry of fMet-tRNA into the A-site enabling it to bind to the 50S P-site, and promotes a ribosome-dependent accommodation of fMet-tRNA into the 70S P-site [50,51]. In *Bacillus subtilis *genetic inactivation of EF-P abolished spore formation without affecting growth [52]. Although it is difficult to predict the effects of EF-P inactivation on erythromycin production, it is relevant to note that erythromycin production in *S. erythraea *is stimulated by streptomycin-resistance mutations [31] and that EF-P was shown to protect 16S rRNA near the G526 streptomycin and the S12 and mRNA binding sites (30S T-site) [50].

In addition to EF-P, other genetic changes occurs in the Px strains affecting the translational machinery, including missense mutations affecting SACE_5926 (*infB*) coding for the translation initiation factor 2, (SACE_0799) (*metS*) coding form methionyl-tRNA synthetase, SACE_0443 (*cysS *paralog) encoding a cysteinyl-tRNA synthatase, SACE_3403 (*gatA*) coding for A subunit of Asp-tRNA Asn/Glu-tRNA Gln amidotransferase, and a frameshift mutation inactivating a paralogous gene (SACE_5919) coding for pseudouridine synthase. These findings emphasize the importance of the translational machinery as a potential target for improvement of antibiotic-producing strains.

The mutations affecting the basic transcription and translation machineries may in turn affect pathway-specific control accounting for the global changes in transcriptional profile, which was observed in Px strain with respect to NRRL 2338 (Figures 4 and 5). As previously mentioned, a total of 459 and 220 genes were up-regulated during growth phase **a **in Px and in NRRL 2338 respectively. Among these DEGs, 138 were up-regulated in both strains, while 335 and 114 were specific for Px and NRRL 2338, respectively (Additional file 3, 4, 5). Within the list of genes specifically up-regulated in the Px strain, we found most of the genes belonging to the *ery *biosynthetic gene cluster. Regulation of the *ery *cluster was found to be altered also in another classically improved *S. erythraea *strain, which exhibited prolonged expression of antibiotic biosynthetic genes compared to the wild type during fermentation [34].

In addition to the ery cluster, SACE_1456 (*mmsA2*) coding for methylmalonate semialdehyde dehydrogenase was also found to be up-regulated in Px. This enzyme provides 6DEB biosynthesis with propionyl-CoA and (2S)-methylmalonyl-CoA precursors (Figure 6) and was found to be up-regulated in a rifampicin-resistant erythromycin-overproducing mutant of *S. erythraea *[32]. Moreover, the expression of *bldD *(SACE_2077), coding for a key developmental regulator that seems to regulate the *ery *cluster positively, was also up- regulated during phase **a **in the Px strain (Figure 8B). Intriguingly, this gene maps immediately downstream of the *pepQ*-*efp*-*nusB *locus in the genome of *S. erythraea *(Figure 8A). In addition to the *ery *cluster and associated CDSs, the genes belonging to the *pks6 *and *tpc5 (geo3) *biosynthetic clusters were also found to be up-regulated in this strain along with *nusA *(SACE_5927 coding for transcriptional termination/antitermination factor NusA), ribosomal protein operons with the associated genes *infA *(SACE_6808 coding for translation initiation factor IF1), *tufA *(SACE_6838 coding for elongation factor EF1A), *fusA *(SACE_6839 coding for translation elongation factor G), and F0F1 ATPase. In contrast, in the list of genes specifically up-regulated in the NRRL 2338, we found many genes related to the TCA cycle including *fumB *(SACE_1784 coding for class I fumarate hydratase), *fumC *(SACE_0930 coding for class I fumarate hydratase), *korA *(SACE_3927 coding for 2- oxaloglutarate ferredoxin oxidoreductase alpha subunit), the urea cycle (*ureA*) (SACE_0634 coding for urease gamma subunit) or the PRPP biosynthesis (*prsA*) (SACE_0816).

In addition to changes in basic transcription and translation machineries, mutations in genes coding for transcriptional factors and signal transduction proteins may contribute to alter pathway-specific control. In particular, 13 genes coding for transcriptional factors and 5 genes encoding two-component system proteins were affected by missense or frameshift mutations in Px (Table 4). However, in the absence of functional data, the effects of such mutations on secondary metabolism may be only hypothesized on the basis of proximity of the mutated regulatory genes to the biosynthetic cluster for secondary metabolites. For instance, SACE_3079, coding for a LysR family transcriptional regulator and affected by a frame shift mutation in the same strain, is genetically linked to a hydrogenase operon (also containing *gmhA *genes, which were shown to be inactivated by frameshift mutations in Px) and may be involved in its regulation. Similarly, SACE_4500, encoding a GntR family transcriptional regulator that is inactivated by a frameshift mutation in Px, maps close to sarcosine oxidase operon. Sarcosine oxidase links glycerophospholipid metabolism to erythromycin biosynthesis catalyzing the oxidative demethylation of sarcosine to glycine, whose metabolism provides the biosynthesis of 6DEB with precursors (Figure 6).

Px strain was obtained by multiple cycle of mutagenesis by chemical (*N*-methyl-*N*-nitro-*N*- nitrosoguanidine) or radiation (X-ray and UV) agents. The sequence data suggests that the mutagenesis/selection process to erythromycin-overproduction phenotype might have been accelerated by selection of mutator phenotype. This hypothesis is supported by missense and frameshift mutations affecting genes involved in DNA replication and repair. In particular, SACE_0826 (*mfd*) coding for transcription/repair coupling factor, SACE_4427 (*mug*) coding for G/U mismatch-specific DNA glycosidase and SACE_5437 (*polA*) were inactivated by frameshift mutations, while missense mutations affected other genes involved in these processes (Table 5).

## Conclusions

Overall our findings demonstrate that the phenotypes of the erythromycin-overproducing strain *S. erythraea *Px are associated with a large number of genetic changes with respect to the reference strain NRRL 2338. Mutations affect 227 CDSs, corresponding to about 3% of CDSs of *S. erythraea *genome. Although certain mutations may be neutral in term of improved antibiotic production, and may have been favored by the mutator background of Px, a considerable number of them map within genes coding for key enzymes involved in central carbon and nitrogen metabolism, and biosynthesis of secondary metabolites, redirecting common precursors toward erythromycin biosynthesis. Several mutations inactivate genes coding for proteins that play fundamental roles in basic transcription and translation machineries including the transcription anti-termination factor NusB and the transcription elongation factor Efp, and genes coding for pleiotropic or pathway-specific regulators, with dramatic effects on global expression profile. The comparison of Px and NRRL 2338 at both genomic and transcriptomic levels not only contributed to elucidate the molecular mechanism underlying the overproduction of erythromycin, but also revealed new possible targets suitable for rationale improvement of industrial antibiotic-producing strains. However, as most of mutated genes are not directly related to the erythomycin over- production, next effort will be to test the genome-assisted predictions by experimental verification.

## Methods

### Bacterial strains and media

*S. erythraea *wild type strain NRRL2338 was a gift of S. Donadio (KtedoGen, Milan, Italy). This strain has been deposited at the American Type Culture Collection. *S. erythraea *Px is an erythromycin-overproducing strain that was obtained by the traditional mutate-and-screen method over a period of about 10 years. The strains were stored in 1-ml cryotubes at -80°C as frozen mycelium in YS medium containing 15% glycerol at a biomass concentration of approximately 0.25 g dry cell weight (DCW) ml^-1^, or at -20°C as spores in 20% glycerol (in distilled water) at a title of approximately 5 × 10^8 ^ml^-1^. The composition (per liter) of the complete media used in this study is reported in Table 8. When requested all media were agarized at a concentration of 1.8%. The composition (per liter) of the nutrient broth agar in the microbiological assays with *Micrococcus luteus *tester strain was: 3 g beef extract, 5 g tryptone, 15 g NaCl, 15 g agar.

**Table 8:** Composition of the media used in this study

Medium	Composition (per liter)	pH
**Complex**		

Seed medium (SM)	4 g peptone, 4 g yeast extract, 2 g KH_2_PO_4_, 4 g K_2_HPO_4_, 0.5 g MgSO_4_7H_2_0, 10 g glucose	7.2

Yeast starch (YS)	2 g yeast extract, 10 g soluble starch	7.3

Oat meal yeast (OMY)	40 g oatmeal, 1 g yeast extract	6.8-7.0

Soluble complete medium (SCM)	20 g soytone, 15 g soluble starch, 10.5 g morpholinepropanesulfonic acid, 1.5 g yeast extract, 0.1 g CaCl_2_	7.2

**Chemically defined**		

MM-101	7 g NH_4_Cl, 3 g KH_2_PO_4_, 7 g K_2_HPO_4_, 0.25 g MgSO_4 _• 7 H_2_O, 0.0138 g CaCl_2 _• 2 H_2_O, 10 g glucose, 2 ml trace solution element (TSE)a	6.9

aWhen indicated 1.0 g casamino acids (Difco, Detroit, Mich.) and/or 2 ml (per liter) trace solution element (TSE) were added. The TSE solution composition (per liter) was: 40 mg ZnCl_2_, 200 mg FeCl_3 _• 6 H_2_O, 10 mg CuCl_2 _• 2 H_2_O, 10 mg MnCl_2 _• 4 H_2_O, 10 mg Na_2_B_4_O_7 _• 10 H_2_O, 10 mg (NH_4_)_6_Mo_7_O_24 _• 4 H_2_O

### Preparation of spores

Concentrated spore suspensions (5 × 10^8 ^ml^-1^) are crucial for purposes like starting reproducible cultures for physiological or fermentation studies. To prepare spores adapted to the conditions of liquid medium, spores were spread on the same medium with agar. Mycelium with spores was strongly attached to the surface agar, thus making impossible to collect spores without agar traces. Therefore, strains have been grown on cellophane discs, as described in [53]. The cellophane discs were sterilized in distilled water and then placed on agar, and the inoculum was spread on cellophane using a glass stick. After two weeks, spores (control in microscope) were easily scraped from cellophane and stored in 20% glycerol at - 20°C.

### Growth conditions

For shake-flask experiments, spores in frozen aliquots were collected by centrifugation, re- suspended in medium 707 (for rehydration), and readily separated by vortexing. Individual aliquots (about 5 × 10^8 ^spores) were used to inoculate each 500 ml baffled Erlenmeyer flask containing 50 ml of the liquid media described above. Cultures were incubated at 30°C with shaking at 250 rpm. Bioreactor cultures were carried out on Minifors mini-fermenters (Infors AG, Bottmingen, CH) that operated with a working volume of 1.5 l. Stirring was provided by Rushton-type impellors rotating at 250 rpm. Sterile air was supplied through a sparger. The bioreactors were equipped with pH electrode, pO2 electrode (polarographic), antifoam probe and Pt-100.

### Erythromycin assays

Erythromycin production in solid media was assayed by bioassay. To this purpose, *S. erythraea *strains were grown in solid media (30 ml) in Petri dishes (8.5 cm). After desired time of cultivation, agar discs of 1.6 cm in diameter (with mycelium on the surface) were removed and placed into empty Petri dishes (diameter 8.5 cm). Petri dishes were then filled with soft nutrient agar seeded with *Micrococcus luteus*. Diameters of the zone of inhibition were measured after 2 days of incubation at 37°C. Agar discs containing defined amounts of > 95% pure erythromycin A (Sigma) were used as a reference. In liquid media, erythromycin was extracted as described [54]. Five hundred μL of *n*-butylacetate were added to 500 μL of broth fermentation, samples were vortexed for 5 min and centrifuged at 9500 g for 15 min. Then the organic phase was mixed with 500 μL buffer 25 mM K_2_PO_4 _pH 5 and centrifuged at 800 g for 5 min. Ten μL of the aqueous phase was injected into HPLC.

HPLC analyses were carried out using an Agilent 1100 Series HPLC system equipped with security guard Cartridges (C18 ODS 4×3 mm) and a Phenomenex-luna 5 μC18 (2) 100 Å column (250×4.6 mm). An isocratic elution mode was used according to [55] Tsuji and Goetz (1978). Mobile phase was as follows: acetonitrile-methanol-0.2 M ammonium acetate-water (45:10:10:35) at pH 7.8, at flow rate of 1 ml min^-1 ^at 25°C using UV detector at 215 nm. Quantification of the erythromycin was achieved using calibration curves of peak area against injected concentration of the various erythromycin standards A, B, C (European Pharmacopoeia HPLC assay).

### DNA procedures

High molecular weight genomic DNA was extracted from *S. erythraea *strains grown in 50 ml of SM medium with shaking at 28°C for 5 days (120 h). After centrifugation, the mycelium was re-suspended in 10 ml SET buffer (75 mM NaCl, 25 mM EDTA, 20 mM Tris- Cl pH 7.5) and incubated in the presence of 5 mg ml^-1 ^lysozyme for 30' at 37°C. Samples were sonicated (Sonifer sonicator Model 250/240, Brain Ultrasonic Corporation) 3 times for 30 sec, and incubated in the presence of 20 mg ml-1 Proteinase K and 1.2% sodium dodecyl sulfate (SDS) for 2 h at 55°C. Nucleic acids were extracted by fenol-chloroform:isoamylic alcohol (24:1) according to standard procedure (Sambrook and Russell, 2001) and RNA removed using 15 μg ml-1 ribonuclease A. After fenol-chloroform:isoamylic alcohol (24:1) extraction and ethanol-precipitation, high molecular-weight DNA was collected by spooling using Shepherd's crooks [56,57].

### Sequencing and assembly of the *S. Erythraea *px genome

Whole-genome shotgun DNA sequencing of *S. erythraea *Px genome was performed by MWG Biotech (Eurofins MWG Operon, Ebersberg, Germany) using frequently cutting restriction enzymes and 2- to 10-kbp fragments cloned into plasmid vectors. Cosmids (32-46 kbp inserts) were also generated from genomic DNA and end-sequenced to provide additional read-pair information, increase coverage of selected regions, and fill the gaps. Remaining gaps and ambiguities were closed using PCR products from specifically designed oligonucleotide primers. Sequence assembly was done using the Phrap assembler43 and editing was done using consed version 14. Repeats were resolved by doing a mini-assembly for the individual sections of the genome and the resulting consensus was integrated into the main genome assembly.

### RNA extraction and RNA microarray experiments

The *S. erythraea *Custom GeneChip used for the NRRL2338 strain gene expression profiling by Peano et al. [24] was used here to analyze the time course gene expression profiling of the Px strain. The sequences of all probes present on the GeneChip were compared with the genomic sequence of the Px strain using BLASTN [58], finding a perfect match for all of them, both for similarity (100%) and length (25 bp).

For each time point, RNA was extracted from mycelium pellets deriving from 1-ml culture samples using the GeneElute™ total RNA Purification Kit (SIGMA), recovering it in 50 μl of Elution Solution. After extraction RNAs were quantified with a NanoDrop spectrophotometer (NanoDrop Technologies) and analyzed by capillary electrophoresis on a Agilent Bioanalyzer (Agilent). The RNA samples showing an RIN (RNA Integrity Number, a quality parameter calculated by the instrument software) value higher than 7 were processed for microarray hybridization, following the instructions for "Prokaryotic Target Preparation" (Affymetrix GeneChip^® ^Expression Analysis Technical Manual). The protocol consists in cDNA synthesis by reverse transcription (starting with 10 μg RNA), followed by cDNA fragmentation with DNase I and labeling with Terminal Deoxynucleotidyl Transferase. The labeled cDNAs were then hybridized for 16 h at 50°C on individual GeneChips. After hybridization, GeneChips were washed and stained with streptavidin-conjugated phycoerythrin by using the Fluidic Station FS450 (Affymetrix) following the FS450_0005 Protocol. Fluorescent images of the microarrays were acquired using a GeneChip Scanner 3000 (Affymetrix). All raw data files are available in Gene Expression Omnibus under accession number GSE30600.

### Genomic comparison and variation detection

The software *Nucmer *from the MUMmer package [59] (http://mummer.sourceforge.net) was used to investigate the presence of possibly large genomic rearrangement. All other comparative analyses have been carried out using the BLAST suite from NCBI (http://www.ncbi.nlm.nih.gov/BLAST,ftp://ftp.ncbi.nih.gov/blast), in particular the BLASTx and BLASTn programs, whose results were then parsed using home-made scripts. The circular plots were generated by an home-made Python script. The sequences of the 227 genes (SACX_) affected by mutations in the Px strain as compared to corresponding genes (SACE_) in the parental NRRL2338 strain have been submitted to GeneBank (accession numbers: from JN392509 to JN392716 in Additional file 7).

### Microarray data analysis

The quality of the raw data obtained from microarray hybridization was assessed considering the MAS5.0 (Microarray Suite/Software, Affymetrix) control parameters after a global scaling at a target intensity of 100. Quality and control parameters as well as box plots of raw intensities highlighted the overall high quality of the data set and the absence of any outlying sample. Probe level data was converted to expression values using both the Robust Multi- array Average (RMA) procedure [60] and the MAS5.0 algorithms. In the former case, PM values (Perfect Match) were background-adjusted, normalized using invariant set normalization, and log transformed. In the latter case, intensity levels were normalized using the Global Scaling option to target value (i.e. TGT = 100).

Genes characterized by a statistically significant modulation of the expression level during the growth time course (*within-class *temporal differential expression) were identified using the EDGE software package, which is based on the Optimal Discovery Procedure [61] and allows identifying genes that are differentially expressed between two or more different biological conditions or to perform significance analysis on time course experiments [62]. Whereas other methods employ statistics essentially designed for testing one gene at a time (e.g. t-statistics and F-statistics), the ODP uses all relevant information from all genes to test each gene for differential expression, thus improving the power of the test. In the particular case of a time course, ODP takes into account the ordering and spacing information provided by the time points.

Briefly, we have tested each gene by first fitting a model (e.g. natural cubic sp-lines) under the null hypothesis that there is no differential expression, and then under the alternative hypothesis that there is differential expression. A statistic is calculated to compare the goodness of fit of the two models under the two different hypotheses. The statistic is a quantification of evidence for transcriptional modulation, and the larger it is the more differentially expressed the gene appears to be. Once the statistic is calculated for each gene, a significance cut-off is applied using a false discovery rate criterion. This process, based on the calculation of the null distribution of the statistics when there is no differential expression, is accomplished through a data re-sampling technique and results in the q-value. Modulated genes are finally selected based on the q-value threshold and, eventually on a fold change limit. Differentially expressed genes were selected considering each time point replicate as an independent entry and setting the confidence threshold at q-value ≤ 0.001. Hierarchical clustering and Eisen's maps were used to group modulated genes and samples in the software package dChip. Before clustering, the expression values for a gene across all samples were standardized (linearly scaled) to have mean 0 and standard deviation 1, and these standardized values were used to calculated correlations between genes and samples and served as the basis for merging nodes. Hierarchical agglomerative clustering was carried out using Pearson correlation coefficient as distance metric and centroid as linkage method.

Chromosomal regions presenting a between-class temporal differential expression were identified using PREDA [35,63,64]. PREDA is a bioinformatic tool developed under R statistical environment for the identification of differentially expressed chromosomal regions, which accounts for variations in gene distance and density. PREDA consists of three main steps: i) computation of a statistic for ranking probes in order of strength of evidence for an expression feature; ii) adaptive bandwidth smoothing of the statistic after sorting the statistical scores according to the chromosomal position of the corresponding genes; and iii) application of a permutation test to identify differentially expressed chromosomal regions with a q-value correction for multiple tests. Transcriptional and structural information are locally integrated smoothing, along the chromosomal coordinate, an expression statistic. The smoothing procedure is approached as a non-parametric regression problem using a local variable bandwidth kernel estimator. A permutation scheme is used to identify differentially expressed regions under the assumption that each gene has a unique neighborhood and that the corresponding smoothed statistic is not comparable with any statistic smoothed in other regions of the genome. The permutation process over B random assignments allows defining a null smoothed statistic for any chromosomal position. The significance of the differentially expressed regions (i.e. the p-value) is computed as the probability that the random null statistic exceeds the observed statistic over B permutations. Once the distribution of empirical p-values has been generated, the q-value is used to identify differentially expressed chromosomal regions according to Storey and Tibshirani [65].

## Competing interests

The authors declare that they have no competing interests.

## Authors' contributions

Conceived and designed the experiments: GDB PA. Performed the experiments: CP AT GC DP SB. Analyzed the data: CP, SB, GDB, PA. Wrote the paper: CP, PA. All authors read and approved the final manuscript.

## Supplementary Material

Additional file 1 **Cell cycle-regulated genes in *S. erythraea *NRRL 2338**. Click here for file

Additional file 2 **Cell cycle-regulated genes in *S. erythraea *Px**. Click here for file

Additional file 3 **Genes up-regulated in both *S. erythraea *NRRL 2338 and *S. erythraea *Px during phase a**. Click here for file

Additional file 4 **Genes specifically up-regulated during phase a in *S. erythraea *Px**. Click here for file

Additional file 5 **Genes specifically up-regulated during phase a in *S. erythraea *NRRL 2338**. Click here for file

Additional file 6 **Growth of *S. erythraea *NRRL 2338 and *S. erythraea *Px on SCM or MM-101 agar with or without amino acid and/or adenine supplement**. Click here for file

Additional file 7 **Accession numbers**. Click here for file
