# Supplementary material for: Comparative genomics and transcriptional profiles of Saccharopolyspora erythraea NRRL 2338 and a classically improved erythromycin over-producing strain
Source: Microb Cell Fact. 2012 Mar 8;11:32. doi: 10.1186/1475-2859-11-32 (PMC3359211; doi:10.1186/1475-2859-11-32)
Supplement: Additional file 6 — Growth of S. erythraea NRRL 2338 and S. erythraea Px on SCM or MM-101 agar with or without amino acid and/or adenine supplement. [file 1475-2859-11-32-S6.PDF]

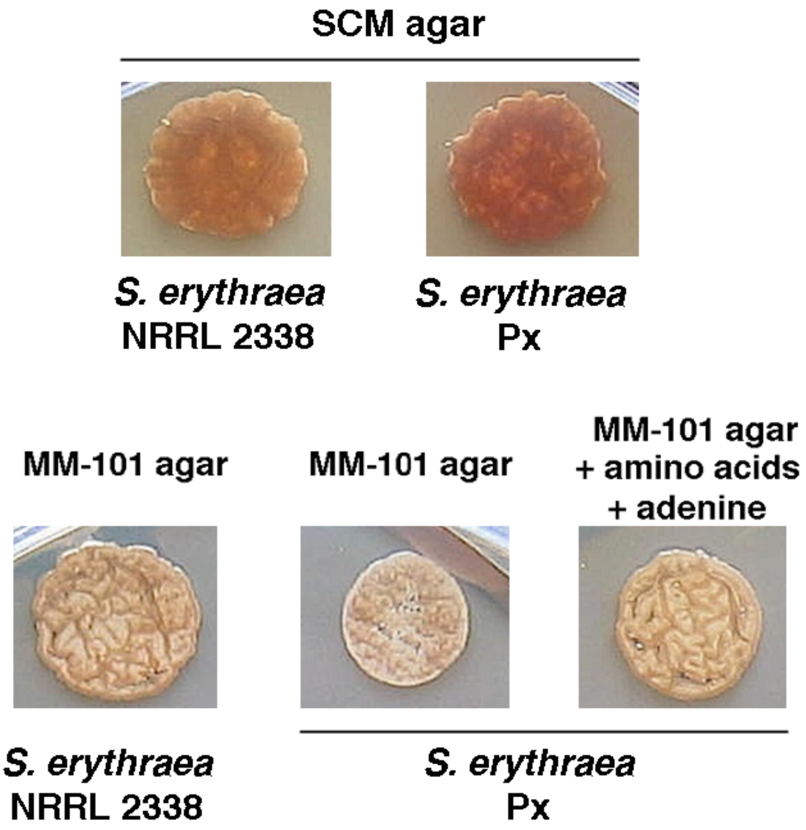

Additional file 1. Growth of *S. erythraea* NRRL 2338 and *S. erythraea* Px on SCM or MM-101 agar with or without amino acid and/or adenine supplement.
