# Supplementary material for: Comparative genomics and transcriptional profiles of Saccharopolyspora erythraea NRRL 2338 and a classically improved erythromycin over-producing strain
Source: Microb Cell Fact. 2012 Mar 8;11:32. doi: 10.1186/1475-2859-11-32 (PMC3359211; doi:10.1186/1475-2859-11-32)
Supplement: Additional file 7 — Accession numbers. [file 1475-2859-11-32-S7.PDF]

Additional file 7. Accession numbers.

SACX_0019 JN392509; SACX_0022 JN392510; SACX_0023 JN392511; SACX_0157 JN392512; SACX_0351 JN392513; SACX_0429 JN392514; SACX_0443 JN392515; SACX_0506 JN392516; SACX_0511 JN392517; SACX_0587 JN392518; SACX_0618 JN392519; SACX_0633 JN392520; SACX_0635 JN392521; SACX_0651 JN392522; SACX_0652 JN392523; SACX_0657 JN392524; SACX_0667 JN392525; SACX_0718 JN392526; SACX_0720 JN392527; SACX_0744 JN392528; SACX_0781 JN392529; SACX_0799 JN392530; SACX_0826 JN392531; SACX_0891 JN392532; SACX_0924 JN392533; SACX_0925 JN392534; SACX_0926 JN392535; SACX_0944 JN392536; SACX_0991 JN392537; SACX_1040 JN392538; SACX_1076 JN392539; SACX_1128 JN392540; SACX_1129 JN392541; SACX_1186 JN392542; SACX_1257 JN392543; SACX_1282 JN392544; SACX_1339 JN392545; SACX_1351 JN392546; SACX_1587 JN392547; SACX_1638 JN392548; SACX_1805 JN392549; SACX_1833 JN392550; SACX_1835 JN392551; SACX_1853 JN392552; SACX_1879 JN392553; SACX_1895 JN392554; SACX_1940 JN392555; SACX_1969 JN392556; SACX_1988 JN392557; SACX_2075 JN392558; SACX_2080 JN392559; SACX_2081 JN392560; SACX_2131 JN392561; SACX_2322 JN392562; SACX_2338 JN392563; SACX_2384 JN392564; SACX_2398 JN392565; SACX_2456 JN392566; SACX_2583 JN392567; SACX_2595 JN392568; SACX_2630 JN392569; SACX_2701 JN392570; SACX_2737 JN392571; SACX_2875 JN392572; SACX_2876 JN392573; SACX_2888 JN392574; SACX_2927 JN392575; SACX_2951 JN392576; SACX_3005 JN392577; SACX_3016 JN392578; SACX_3033 JN392579; SACX_3038 JN392580; SACX_3057 JN392581; SACX_3069 JN392582; SACX_3071 JN392583; SACX_3073 JN392584; SACX_3079 JN392585; SACX_3102 JN392586; SACX_3132 JN392587; SACX_3186 JN392588; SACX_3187 JN392589; SACX_3216 JN392590; SACX_3262 JN392591; SACX_3264 JN392592; SACX_3346 JN392593; SACX_3348 JN392594; SACX_3361 JN392595; SACX_3366 JN392596; SACX_3403 JN392597; SACX_3450 JN392598; SACX_3478 JN392599; SACX_3524 JN392600; SACX_3579 JN392601; SACX_3677 JN392602; SACX_3737 JN392603; SACX_3745 JN392604; SACX_3748 JN392605; SACX_3756 JN392606; SACX_3800 JN392607; SACX_3850 JN392608; SACX_3925 JN392609; SACX_3936 JN392610; SACX_3961 JN392611; SACX_3978 JN392612; SACX_4034 JN392613; SACX_4066 JN392614; SACX_4072 JN392615; SACX_4140 JN392616; SACX_4249 JN392617; SACX_4288 JN392618; SACX_4307 JN392619; SACX_4310 JN392620; SACX_4319 JN392621; SACX_4325 JN392622; SACX_4347 JN392623; SACX_4420 JN392624; SACX_4427 JN392625; SACX_4434 JN392626; SACX_4451 JN392627; SACX_4454 JN392628; SACX_4500 JN392629; SACX_4536 JN392630; SACX_4560 JN392631; SACX_4563 JN392632; SACX_4589 JN392633; SACX_4596 JN392634; SACX_4651 JN392635; SACX_4775 JN392636; SACX_4854 JN392637; SACX_4937 JN392638; SACX_4982 JN392639; SACX_5012 JN392640; SACX_5030 JN392641; SACX_5053 JN392642; SACX_5073 JN392643; SACX_5147 JN392644; SACX_5196 JN392645; SACX_5208 JN392646; SACX_5255 JN392647; SACX_5284 JN392648; SACX_5285 JN392649; SACX_5286 JN392650; SACX_5291 JN392651; SACX_5299 JN392652; SACX_5301 JN392653; SACX_5308 JN392654; SACX_5311 JN392655; SACX_5410 JN392656; SACX_5423 JN392657; SACX_5425 JN392658; SACX_5427 JN392659; SACX_5430 JN392660; SACX_5435 JN392661; SACX_5437 JN392662; SACX_5446 JN392663; SACX_5460 JN392664; SACX_5482 JN392665; SACX_5483 JN392666; SACX_5513 JN392667; SACX_5523 JN392668; SACX_5734 JN392669; SACX_5739 JN392670; SACX_5741 JN392671; SACX_5787 JN392672; SACX_5905 JN392673; SACX_5919 JN392674; SACX_5926 JN392675; SACX_5955 JN392676; SACX_6021 JN392677; SACX_6086 JN392678; SACX_6087 JN392679; SACX_6104 JN392680; SACX_6108 JN392681; SACX_6118 JN392682; SACX_6133 JN392683; SACX_6184 JN392684; SACX_6246 JN392685; SACX_6319 JN392686; SACX_6323 JN392687; SACX_6326 JN392688; SACX_6330 JN392689; SACX_6385 JN392690; SACX_6416 JN392691; SACX_6447 JN392692; SACX_6490 JN392693; SACX_6564 JN392694; SACX_6567 JN392695; SACX_6578 JN392696; SACX_6588 JN392697; SACX_6664 JN392698; SACX_6668 JN392699; SACX_6681 JN392700;

SACX_6720 JN392701; SACX_6764 JN392702; SACX_6765 JN392703; SACX_6773 JN392704; SACX_6784 JN392705; SACX_6927 JN392706; SACX_6961 JN392707; SACX_6971 JN392708; SACX_6972 JN392709; SACX_7047 JN392710; SACX_7125 JN392711; SACX_7193 JN392712; SACX_7202 JN392713; SACX_7240 JN392714; SACX_7243 JN392715; SACX_7263 JN392716
